# Pathological characteristics of a murine oral coxsackievirus A10 infection model

**DOI:** 10.1128/jvi.00937-25

**Published:** 2025-07-01

**Authors:** Jichen Li, Tianjiao Ji, Qian Yang, Guoyan Zhang, Wei Duan, Rui Wang, Ying Liu, Huijie Li, Qiang Sun, Jianfang Zhou, Yong Zhang

**Affiliations:** 1 National Key Laboratory of Intelligent Tracking and Forecasting for Infectious Diseases (NITFID), National Institute for Viral Disease Control and Prevention, Chinese Center for Disease Control and Prevention 12415, Beijing, China; 2 National Laboratory for Poliomyelitis, WHO WPRO Regional Polio Reference Laboratory, National Institute for Viral Disease Control and Prevention, Chinese Center for Disease Control and Prevention 12415, Beijing, China; 3 National Health Commission Key Laboratory of Microbial Genomics, National Health Commission Key Laboratory for Biosafety, National Institute for Viral Disease Control and Prevention, Chinese Center for Disease Control and Prevention, Beijing, China; 4 Beijing Dongcheng District Center for Disease Control and Prevention (Beijing Dongcheng District Health Supervision Institute), Beijing, China; University of Kentucky College of Medicine, Lexington, Kentucky, USA

**Keywords:** CVA10, oral infection, ICR mice model, RNA-seq

## Abstract

Coxsackievirus A10 (CVA10) is an enterovirus that has caused global outbreaks of hand, foot, and mouth disease (HFMD), accompanied by neurological and systemic complications. Animal models that can simulate natural infections are necessary for studying viral pathogenesis. In this study, our objective was to establish a mouse-adapted strain (CVA10-P8) through serial passaging, which was capable of orally infecting 14-day-old ICR mice, leading to hind-limb paralysis and death. Viral titers in various tissues indicated tropism in muscle tissue, with significant increases observed in the brain, lung, and intestinal tissues as the infection progressed. Pathological examination revealed tissue damage in the muscles, brain, lungs, and intestines accompanied by neutrophil infiltration of the brain. Furthermore, flow cytometry and transcriptome analysis revealed metabolic abnormalities, immune system activation, and the promotion of systemic inflammatory responses in the brain, muscle, and lung tissues. In summary, we successfully developed a CVA10 mouse-adapted strain and a corresponding mouse model, providing valuable tools for studying CVA10 pathogenesis and evaluating the efficacy of antiviral interventions.

**IMPORTANCE:**

CVA10 has emerged as a predominant pathogen in the etiology of HFMD, with the potential to elicit neurological manifestations and systemic complications. In this study, we successfully established a novel murine model of CVA10 infection by serially propagating a clinical isolate of CVA10, which enabled oral infection in 14-day-old ICR mice. This model facilitated the investigation of the pathogenesis of CVA10-induced disease. Utilizing this infection model, we employed flow cytometry and transcriptome analysis to elucidate the central nervous system (CNS) inflammatory responses elicited by CVA10 in mice, which closely mimic the natural route of infection. Our findings provide novel insights into the pathophysiological mechanisms underlying CVA10-induced neuroinflammation and pave the way for further research into targeted therapeutic interventions for HFMD associated with CVA10.

## INTRODUCTION

Over the past decade, enterovirus A71 (EV-A71), coxsackievirus A16 (CVA16), coxsackievirus A6 (CVA6), and coxsackievirus A10 (CVA10) have emerged as the primary pathogens causing hand, foot, and mouth disease (HFMD) (1–3). Research indicates that EV-A71 and CVA16 are predominantly responsible for global outbreaks of this condition (4–6). Notably, the prevalence of CVA10 has increased in mainland China (7). CVA10 belongs to the *Enterovirus* genus of the *Picornaviridae* family, specifically the EV-A group (8). Although most cases associated with CVA10 infection exhibit self-resolving symptoms, the virus can occasionally cause severe complications, including acute flaccid paralysis, aseptic meningitis, and in rare instances, even death (9–12).

During the HFMD outbreak in Wuhan between 2012 and 2013, the detection rate of CVA10 increased to 41% (13). In 2018, CVA10 was identified in 25% of the cases in Guangzhou (14). These issues also pose a challenge to the prevention and control of HFMD in China (15). Nevertheless, studies on the infection mechanisms of CVA10 are scarce, and the market is devoid of specific medications and vaccines. Therefore, developing relevant animal models can help improve our understanding of the pathogenic mechanisms of CVA10 (16–18).

Animal models have played an important role in elucidating the pathogenic mechanisms of CVA10 and evaluating the effectiveness of antiviral treatments (19–21). At present, the main infection routes in these animal models are intraperitoneal injection, intramuscular injection, and intracranial injection, which deviate from the natural infection routes (19, 22, 23). This discrepancy may limit the applicability of research on CVA10. Owing to the species-specific nature of human enteroviruses, infecting mice through the oral pathway is challenging. Therefore, our goal was to develop an oral infection model of 14-day-old ICR mice using a mouse-adapted CVA10 strain. This model enabled us to analyze tissue tropism and pathological features of the virus, thereby enhancing our understanding of its pathogenicity. Using this model, we evaluated the molecular changes induced by CVA10 in mice and further elucidated the molecular mechanisms underlying CVA10 infection.

## RESULTS

### Establishment of the CVA10-P8 infection model

**Fig 1:**
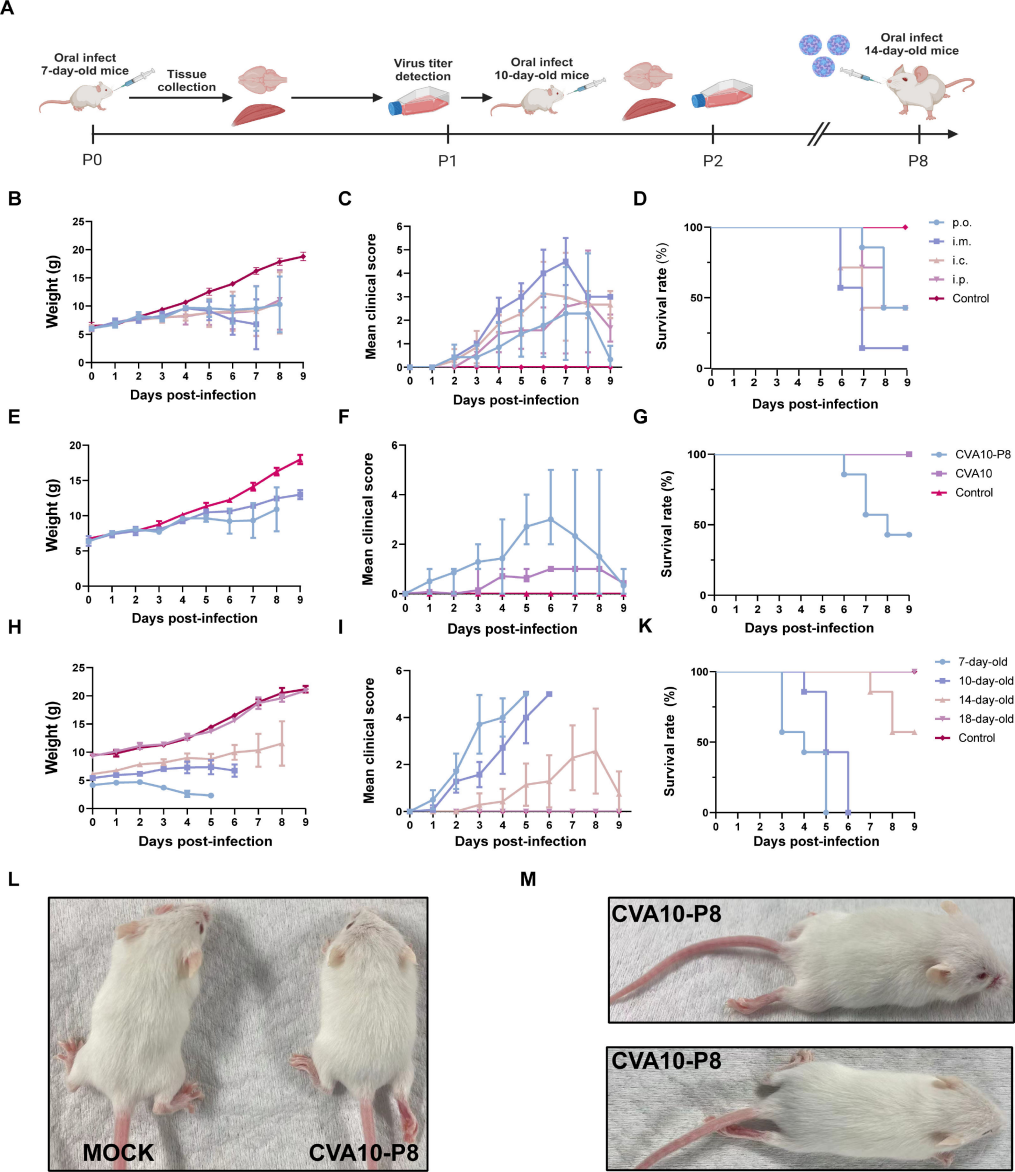
Establishment of the mouse-adapted strain and virus infection mouse model. The CVA10-P8 was serially passaged in ICR mice via p.o. (A). Body weight, survival rate, and clinical scores of 14-day-old mice inoculated via the i.c., i.p., i.m., and p.o. (B–D) were monitored daily; control mice were inoculated with CVA10 strain SZK2021GY4/89 or uninfected culture medium via p.o. (E–G); ICR mice aged 7, 10, 14, and 18 days were infected orally with 10^6^ TCID_50_ (H–K). Clinical scores of CVA10-P8 infected and control mice. 0, no disease; 1, ruffled fur; 2, weight loss; 3, single-limb paralysis; 4, paralysis of both hindlimbs; 5, moribund or dead. Clinical symptoms of CVA10-P8 infection in 14-day-old mice at 6 dpi (L, M).

We successfully adapted CVA10 such that it was able to infect 14-day-old ICR mice via the oral (p.o.) route (Fig. 1A). To investigate the clinical symptoms and mortality associated with various infection pathways in mice, we inoculated mice with the adapted strain (CVA10-P8) via the intracranial (i.c.), intramuscular (i.m.), intraperitoneal (i.p.), and p.o. routes. Our results showed that all injections led to significant weight loss in infected mice (Fig. 1B). Mice infected with CVA10-P8 via the p.o. route began to show symptoms at 6 dpi, such as hind-limb paralysis and death (Fig. 1L and M), but other injection routes had a lower proportion of these clinical symptoms (Fig. 1C and D). Although infected mice via i.m. exhibited higher clinical scores as well as mortality, the p.o. infection more closely mimicked natural infection (Fig. 1D). After p.o. injection of 14-day-old ICR mice with 10^6^ TCID_50_ of CVA10-P8, distinct clinical symptoms appeared at 6 dpi, and by 9 dpi, over 50% of the mice succumbed to infection. However, these symptoms were not observed in the CVA10 strain (Fig. 1E through G). To compare the susceptibilities of mice of different ages, 7-, 10-, 14-, and 18-day-old ICR mice were inoculated with CVA10-P8. The results indicated that 7- and 10-day-old mice infected with CVA10-P8 died 5–6 dpi, whereas 18-day-old mice withstood the challenge with CVA10-P8, exhibiting no weight loss, hind-limb paralysis, or mortality (Fig. 1H through K). These results showed that CVA10-P8 was adapted to 14-day-old mice via p.o. injection.

### Dynamic change in viral titers in mouse tissues

**Fig 2:**
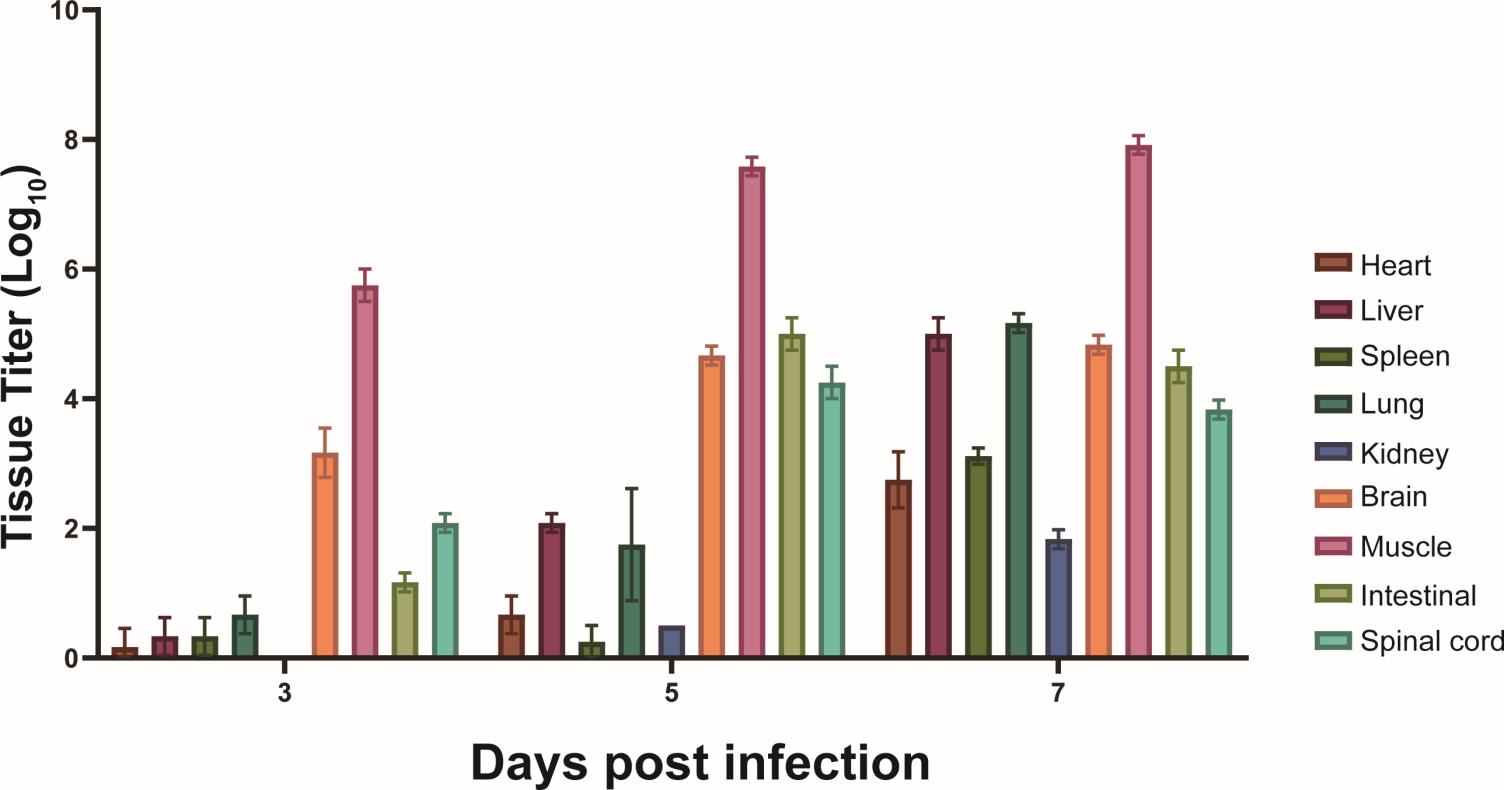
Viral titers in tissues from 14-day-old mice infected with CVA10-P8. CVA10-P8 was inoculated into 14-day-old ICR mice. Mice were euthanized by carbon dioxide after 3, 5, and 7 days, and the viral titers were measured in each tissue. Viral titers in the heart, liver, spleen, lung, kidney, brain, muscle, intestinal, and spinal cord at each time point represent the average data from seven mice. Data are shown as the mean ± SD. ^**^P < 0.01; ^***^P < 0.001; ^****^P < 0.0001; ns, not significant.

We investigated viral dissemination in 14-day-old ICR mice following infection with CVA10-P8 using viral titers in mouse tissue. Virus was detected in the early stages (3 dpi) of infection in the heart, liver, spleen, lungs, kidneys, brain, skeletal muscle, intestine, and spinal cord. Notably, at 3 dpi, exceptionally high viral titers were observed in skeletal muscles, suggesting they serve as primary early replication sites for the virus. At 5 dpi, viral titers in all tissues increased although virus was still most prominent in the muscle, brain, spinal cord, skeletal muscle, and intestine. Between 5 and 7 dpi, there was no substantial increase in titer in the muscle, brain, spinal cord, and intestine, but titer did increase in the other tissues. In the skeletal muscle, which consistently had the highest viral titers, the viral titer reached 10^8^ TCID_50_ at 7 dpi, and the mice exhibited severe hind-limb paralysis (Fig. 2).

### CVA10-P8 leads to pathological changes in mouse tissues and neutrophil recruitment in the brain

**Fig 3:**
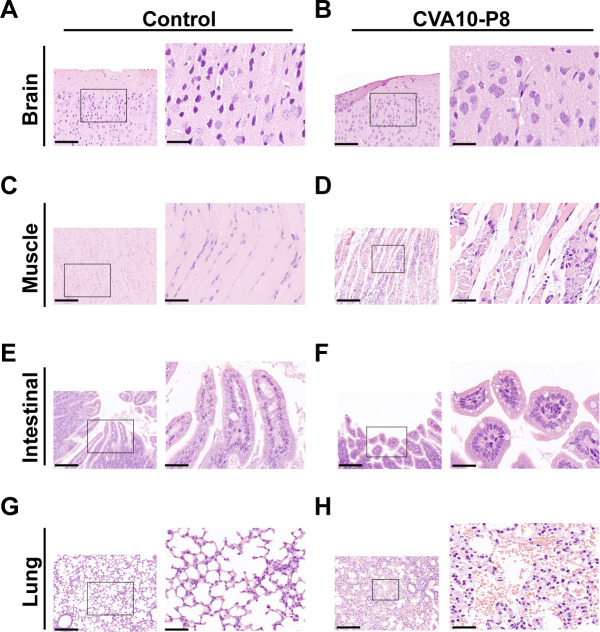
Histopathological changes in 14-day-old mice infected via p.o. with 10^6^ TCID_50_. Histopathological examination was performed on brain, muscle, intestinal, and lung tissue from infected mice (B, D, F, and H) using H&E, control mice received uninfected culture medium (A, C, E, and G). Magnification: left, 100×, right, 400×. Images are representative of the results obtained in three mice with the same clinical symptoms.

To further delineate the pathogenesis of CVA10-P8 under conditions mimicking natural infection, we conducted hematoxylin and eosin (H&E) staining of tissues with elevated viral titers in the brain (Fig. 3A and B), skeletal muscle (Fig. 3C and D), intestine (Fig. 3E and F), and lungs (Fig. 3G and H). In 14-day-old ICR-infected mice, pathology escalated with time, notably manifesting as pronounced fiber degeneration and fragmentation within the skeletal muscle (Fig. 3D). During the late stage of infection, the intestinal mucosa underwent partial denudation accompanied by substantial villous sloughing and a decrease in height, leading to structural disorganization (Fig. 3F). The pulmonary tissue sustained severe injury characterized by extensive disruption of alveolar structures. Pronounced infiltration of hematopoietic and inflammatory cells was observed within the interstitial and alveolar spaces (Fig. 3H).

**Fig 4:**
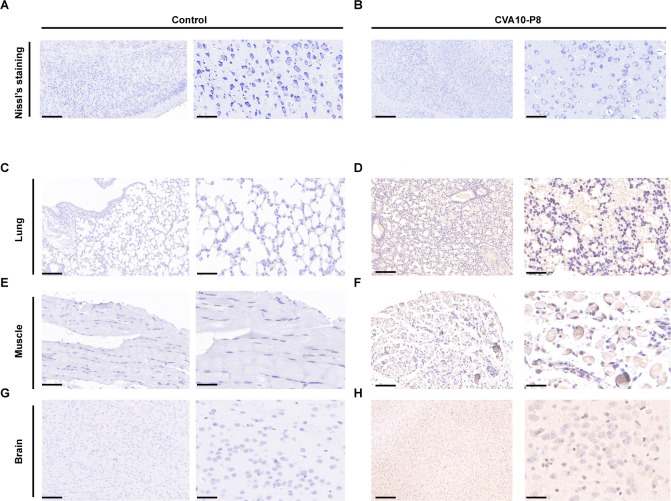
CVA10-P8 infection induces brain injury and distribution of viral antigens in tissues. Nissl’s staining analysis revealed diminished or absent Nissl bodies, and nuclear vacuolation (**A, B**). IHC was used to analyze the distribution of viral antigens in the lung (**C, D**), hind limbs (**E, F**), and brain (**G, H**). left, 200×; right, 400×.

To further elucidate the neuropathological alterations associated with CVA10-p8 infection, a histopathological examination of the brain tissue was performed (Fig. 3B). The results showed interstitial rarefaction within the cerebral parenchyma, neuronal cytomegaly, a pronounced decrease in neuronal density, and the detection of viral antigens in brain tissue sections, indicating that CVA10 penetrated the central nervous system (CNS) (Fig. 4A, B, G and H). In addition, the substantial deposition of viral antigens in both lung and muscle tissues indicated that CVA10 replicates in 14-day-old mice via the p.o. route and resulted in tissue damage, suggesting a systemic pathogenic effect of the virus on ICR mice (Fig. 4C through F).

**Fig 5:**
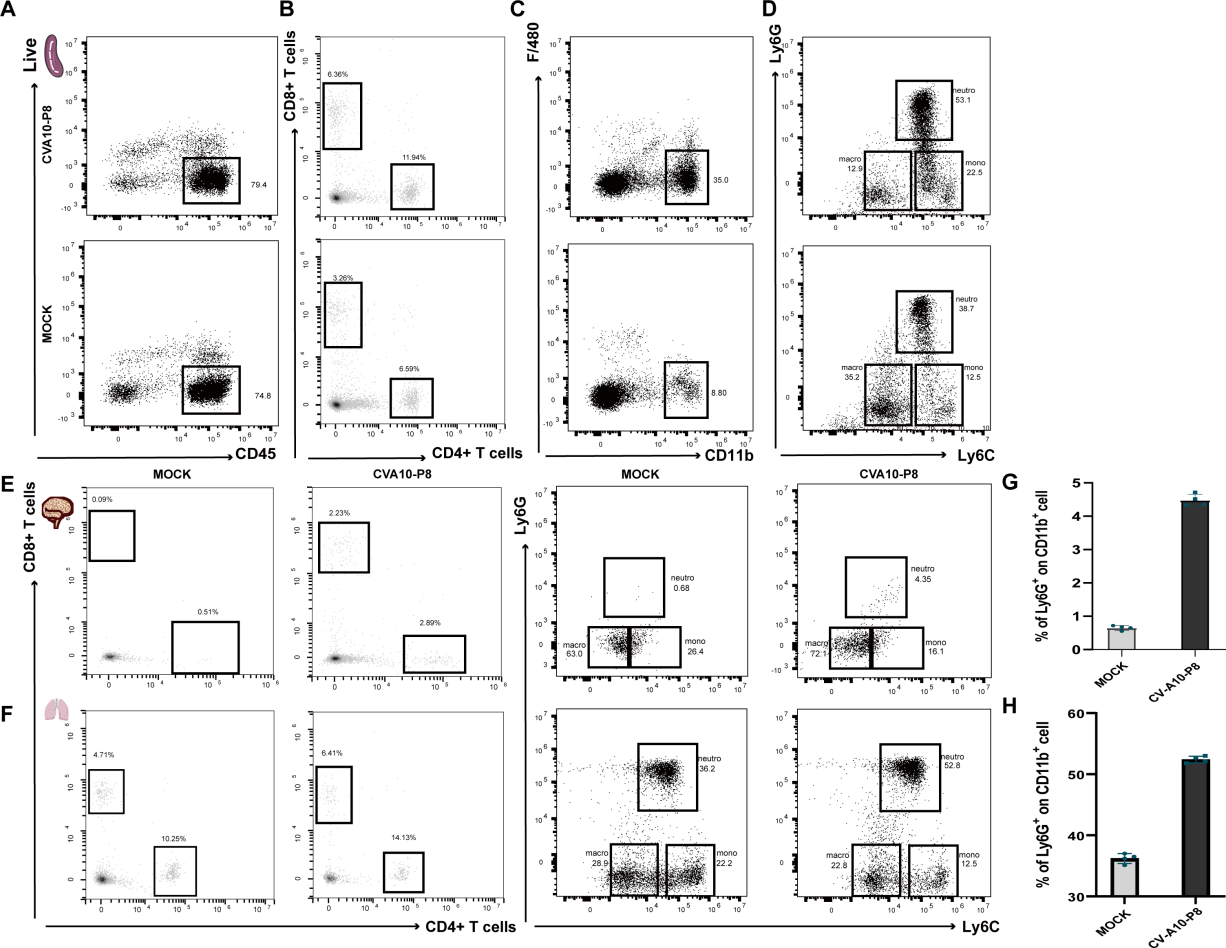
Phenotypes of leukocytes in infected mouse tissue. The proportions of CD45^+^, CD4^+^ CD8^+^ T cells, CD11b^+^, and CD11b^+^Ly6G^+^ cells in the spleen (**A, B, C, and D**), CD4^+^ CD8^+^ T cells, CD11b^+^Ly6G^+^ cells in the brain (**E**) and lungs (**F**). The number of inflammatory cells in the brain and lungs (**G, H**). Statistical results of lymphocytes, neutrophils, monocytes, and macrophages in the brain and lung; *n* = 4 per group. Data are shown as the mean ± SD. ^**^P < 0.01; ^***^P < 0.001; ^****^P < 0.0001; ns, not significant.

To explore the impact of CVA10-p8 infection on immune homeostasis, we used fluorescence-activated cell sorting to examine the phenotypes of immune cells in the spleen (Fig. 5A through D), lungs (Fig. 5F and H), and brain tissues (Fig. 5E and G). The ratios of CD4^+^ CD8^+^ (lymphocytes), CD11b^+^F480^+^ (neutrophil precursor), and CD11b^+^Ly6G^+^ (neutrophil) cells are significantly elevated after viral infection (24). We observed a marked increase in the number of neutrophils in the brain tissue of the infected mice(Fig. 5E and G), suggesting that viral infection may increase the permeability of the blood–brain barrier, facilitating the infiltration of neutrophils.

### CVA10-P8 infection caused a systemic inflammatory response at the transcriptome level

**Fig 6:**
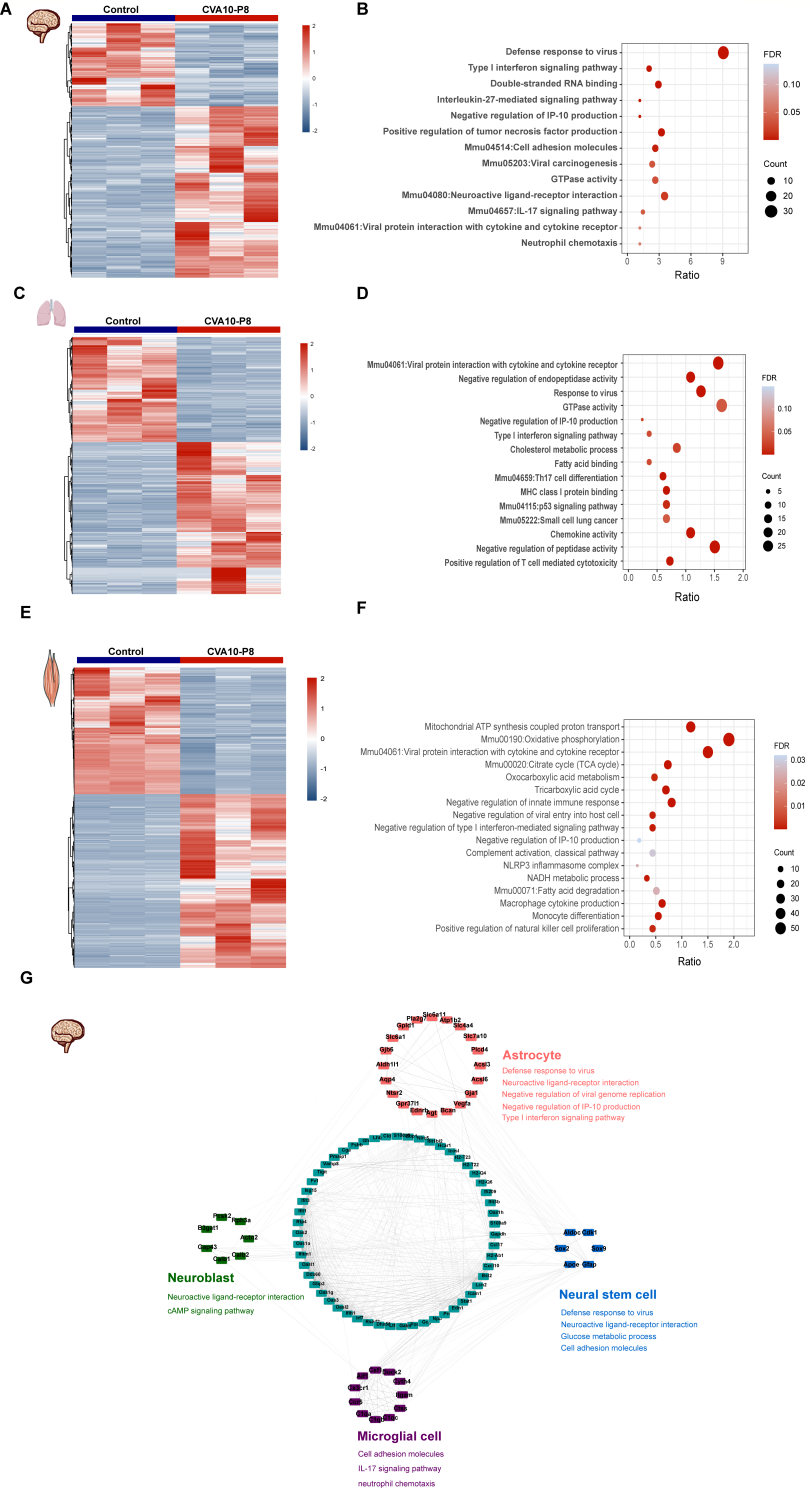
Transcriptome analysis of the brain, lung, and skeletal muscle tissues from 14-day-old mice. Heat maps of differentially expressed genes (DEGs) in the skeletal muscle and brain tissue. (A, C, and E) Gene Ontology and Kyoto Encyclopedia of Genes and Genomes enrichment analysis of DEGs in tissues; (B, D, and F) Correlation between cell type marker proteins and DEGs in the brain. Cell type and related marker proteins are listed in (G). Cell types and related marker proteins (red, blue, green, and purple) are generalized. The correlation between cell type marker proteins and DEGs in the brain (dark green) is data specific.

To further understand the biological processes and pathways through which CVA10-P8 causes pathological damage in mouse tissues, we collected the brain, lung, and muscle tissues for transcriptome analyses. The heatmap results of tissues from infected mice suggested that infection with CVA10-P8 induced significant differential gene expression (Fig. 6A, C and E). Gene Ontology (GO) and Kyoto Encyclopedia of Genes and Genomes (KEGG) enrichment analyses revealed that numerous differentially expressed genes (DEGs) in the muscle tissues were enriched in pathways related to viral protein interactions with cytokines and cytokine receptors, oxidative phosphorylation, macrophage cytokine production, and the tricarboxylic acid cycle (Fig. 6F). In the lung tissue, genes were enriched in response to the virus, type I interferon signaling pathway, chemokine activity, and fatty acid binding, which also suggested severe damage to the lung tissue by the virus (Fig. 6D). Furthermore, in the brains of CVA10-P8-infected mice, the enriched pathways were associated with the defense response to the virus, neuroactive ligand-receptor interaction, IL-17 signaling pathway, and neutrophil chemotaxis (Fig. 6B).

Considering the metabolic heterogeneity among different neuronal cell types, we subsequently analyzed cell-specific markers and protein-protein interaction networks to identify cell-specific metabolic changes (Fig. 6G). We found that the IL-17 signaling pathway and neutrophil chemotaxis occur in microglial cells, and the cAMP signaling pathway was observed in neuronal progenitor cells. Neuroactive ligand-receptor interaction, defense response to viruses, and type I interferon pathways were evident in astrocytes and neural stem cells (Fig. 6G).

## DISCUSSION

Although the majority of enteric viral infections are self-limiting and asymptomatic, a subset of infections can present with severe manifestations, including HFMD, myocarditis, aseptic meningitis, and respiratory diseases (25, 26). Over the past decade, non-polio enteric viruses have emerged as major public health concerns (27). CVA10 has been identified as a prominent etiological agent in the pathogenesis of HFMD (11, 28). Additionally, CVA10 has demonstrated the capacity for co-infection and genetic recombination with other enteric viruses, such as EV-A71, CVA16, and CVA6 (4, 29, 30), further complicating disease dynamics and public health strategies.

Animal models are an efficacious research instrument for elucidating the pathogenic mechanisms of viral infections, assessing the efficacy of vaccines, and evaluating therapeutic antibodies (31–33). In previous studies, the successful establishment of EV-A71 infection models in rhesus macaques, neonatal mice, and immunocompromised mice significantly advanced the study of EV-A71 infections (34–36). The human KREMEN-1 receptor has a high degree of identity with the mouse ortholog, this HFMD agent can be more easily studied than those Enterovirus A types which have essential receptors which do not have murine orthologs which can interact with the human virus. Previous researchers have successfully established a CVA6 strain that can infect 10-day-old ICR mice via oral route (23).

In this study, we successfully adapted a CVA10 mouse-adapted strain for p.o. infection in 14-day-old ICR mice, thereby mimicking the natural infection of human to a certain degree. In our previous studies, we observed that some enteroviruses exhibited higher titers in mouse muscle tissue compared to other tissues despite not being administered via i.m. This discrepancy may arise from the virus’s distinct tropisms for different tissues. Although oral administration first introduces the virus to the intestinal tract, its replication efficiency within the intestinal tissue is relatively lower than in muscle tissue. As a unique organ in the body, the brain is protected by two systems of defense and homeostasis, with the blood-brain barrier (BBB) being a critical component (37). While the BBB plays a vital role in shielding the brain from viral invasion, it also serves as a key interface for enterovirus to breach into the central nervous system (38, 39). Our findings suggest that the CVA10-P8 strain exhibits a strong tropism for muscle tissue, where it preferentially replicates. Central nervous system infections by this strain may, therefore, occur as a secondary consequence of muscle tissue infection. Furthermore, we conducted a comprehensive assessment of the pathological damage caused by CVA10 infection. Examination via H&E staining and immunohistochemistry (IHC) of various tissues revealed that CVA10 infection resulted in the disruption of muscle fibers, substantial leukocytic infiltration in pulmonary tissues, and diminution of neuronal cells within the brain. These observations were consistent with the viral titers detected in the respective tissues. Additionally, we observed that in the early stages of infection, the virus replicated extensively in muscle tissues. As the disease progressed, the viral titer significantly increased in the lungs and intestines, which may correspond to the clinical symptoms associated with CVA10. Our findings demonstrate that the muscular tissue is the most active site for viral replication. Concurrently, pronounced pathological alterations were observed in the pulmonary, cerebral, and intestinal tissues. The detection of viral antigens within the brain, coupled with the observed reduction in neuronal cell populations, suggests active infection with CVA10 targeting the CNS of mice. Given the high conservation of the human KREMEN-1 receptor in mice, this enterovirus type associated with hand, foot, and mouth disease is more suitable for study compared to other enterovirus types. But the CVA10 mouse- adapted strain we constructed and the previously reported CVA6 mouse-adapted strain may utilize the KREMEN-1 receptor through different mechanisms. After comparing the differences in nucleotide and amino acid sequences between the mouse-adapted and parental strains, we observed that the mutation sites in the CVA10 mouse-adapted strain did not overlap with the previously reported KREMEN-1-binding domain (Fig. S1; Table 1) (40, 41). However, the CVA10 mouse adapted strain exhibited higher viral titers than the parental strain in SK-N-SH cells (a cell line insensitive to CVA10) at different time points, which leads us to hypothesize that there may be unknown receptors or host factors that contribute to the generation of CVA10 mouse adapted strain (Fig. S2). This may also potentially explain the more severe pathological damage observed in elderly mice.

**TABLE 1:** Nucleotide differences between the sequences of the CVA10 parental strain and the CVA10 adapted strain

Region	Nucleotide			Amino acid		
	Position	P0	P8	Position	P0	P8
VP4	193	T	A	65	S(TCA)	T(ACA)
VP2	246	C	T	82	NC	
VP2	394	A	G	132	I(ATC)	V(GTC)
VP3	172	A	G	58	T(ACC)	A(GCC)
VP3	319	G	A	107	A(GCA)	T(ACA)
VP1	139	C	T	47	NC	
2C	853	A	G	285	S(AGT)	G(GGT)
3D	1053	T	C	351	NC	

We extended our investigation through comprehensive transcriptomic analysis to elucidate the systemic immune response elicited by CVA10 infection. Viruses can reprogram metabolic pathways in host cells, thereby facilitating their rapid replication. This metabolic reprogramming can precipitate bioenergetic stress and apoptosis in host cells. Our analysis of transcriptomic data from the lung, brain, and muscle tissues of infected mice revealed a significant enrichment of DEGs associated with the tricarboxylic acid cycle, GTPase activity, fatty acid binding, and cholesterol metabolic processes. These metabolic alterations may facilitate viral entry, assembly, and release (42, 43). Following viral infection, there is marked enrichment in activated immune pathways and inflammatory responses (44). These genes augment the acquired immune response by engaging effector cells. Notably, viral infections also elicit an inflammatory response in the brain (45, 46). Our observations indicate an enrichment of neutrophil chemotaxis and the IL-17 signaling pathway within the brain tissue, which corroborates the results obtained from flow cytometry analysis.

Enteroviral infections can spread from the initial site of infection to the CNS. Our results suggest that CVA10-P8 infection can induce the infiltration of inflammatory cells within the brain, potentially leading to a disruption in the integrity of the blood–brain barrier. In summary, these results mimic the natural infection of mice with CVA10 and exhibit neuropathological characteristics and damages akin to clinical features, thereby providing a valuable tool for investigating the pathogenesis of CVA10 and for evaluating the efficacy of vaccines and antivirals.

## MATERIALS AND METHODS

### Cell and virus

Human rhabdomyosarcoma (RD, ATCC, USA) cell lines were maintained in minimum essential medium containing 10% fetal bovine serum (Gibco, USA) at 37°C in a 5% CO_2_ humidified incubator. The CVA10 strain SZK2021GY4/89 was isolated from Gansu Province, China. The 7-day-old ICR mice were infected with the CVA10 strain via the p.o. route and monitored daily for clinical signs. Upon the onset of hind-limb paralysis in mice, brain and muscle tissues were collected, homogenized in PBS solution with 1% penicillin-streptomycin (HyClone, USA), and then centrifuged at 10,000 *g* for 10 min. The supernatant was collected and used to infect RD cells. Once cytopathic effects were observed, the mixture was subjected to three rounds of freeze–thawing and subsequently filtered through a 0.22 µm filter to yield the purified supernatant. The supernatant was then inoculated via p.o. into 7-, 10-, or 14-day-old ICR mice. Finally, we successfully generated a CVA10 strain (CVA10-P8) that was adapted to infect 14-day-old ICR mice through the p.o.

### Establishment of the neonatal murine model of CVA10 infection

Fourteen-day-old ICR mice were infected with CVA10-P8 through the i.c., i.m., i.p., and p.o. routes (*n* = 7 per group); 7-, 10-, 14-, and 18-day-old ICR mice were inoculated with CVA10-P8 for age-dependent experiments. The control group was inoculated with an uninfected culture medium. Weights, survival rates, and clinical scores of the mice were recorded daily. The grade of the clinical disease was scored as follows: 0, no disease; 1, ruffled fur; 2, weight loss; 3, single-limb paralysis; 4, paralysis of both hindlimbs; and 5, moribund or dead.

### Dynamic monitoring of CVA10 viral titers in mouse tissues

The 14-day-old ICR mice were infected with CVA10-P8 at 10^6^ 50% tissue culture infective dose (TCID_50_) through the p.o. route (*n* = 7 per group). Following euthanasia, the heart, liver, spleen, lung, kidney, brain, skeletal muscle, intestine, and spinal cord were collected at 3, 5, and 7 dpi. All tissue supernatants underwent 10-fold serial dilution and were inoculated into RD cells cultured in 96-well plates. After 7 days of continuous observation, viral titers were detected using the TCID_50_ assay.

### Histopathological and immunohistochemistry assay

After p.o. injection of the mouse-adapted CVA10 strain (CVA10-P8) and CVA10 strain (SZK2021GY4/89) into 14-day-old ICR mice, the brain, muscles, intestines, and lungs of the experimental and control groups were harvested. The tissues were fixed in formalin buffer for 24 h, dehydrated, embedded in paraffin, and sliced into 5 µm thick sections. H&E staining was performed after dewaxing the tissue sections with xylene. The brain tissue was cut into 5 μm sections, and Nissl staining was performed to detect the surviving neurons.

In the IHC experiment, the brain, muscle, and lung tissues of ICR mice were dewaxed, dehydrated, and microwaved for 10 min for antigen repair. The tissue was sealed with a 3% bovine serum albumin blocker and incubated with mouse polyclonal anti-VP1 CVA10 (1:100 dilution, ABclonal). Next, a horseradish peroxidase-labeled secondary antibody (1:1,000 dilution) was added and incubated for 50 min at room temperature (20°C). Finally, all images were observed using AxioCam MRc5 (Carl Zeiss, Berlin, Germany) at magnifications of 200× or 400×.

### Library preparation for transcriptome sequencing

Total RNA was extracted from the brain, muscle, and lung tissues, including from the control group infected with CVA10-P8 (*n* = 3 per group), using TRIzol reagent. RNA concentrations were determined using a Qubit 2.0 fluorometer (Invitrogen, USA), and RNA integrity, with a minimum RIN value of 8, was evaluated using a Bioanalyzer 2100 (Agilent, USA). The first strand of cDNA was synthesized via random primer reverse transcription, and the second strand was amplified using RNase H and DNA polymerase. The resulting cDNA was enriched and purified before sequencing on an Illumina NovaSeq 6000 platform. Gene expression levels were measured using feature count software (version 1.5.0).

### RNA-seq data analysis

Reads with perfect matches or single mismatches were selected for downstream analysis and annotation of the *Homo sapiens* reference genome. The Hisat2-StringTie-Edge R pipeline was used for read mapping, transcript assembly, and identification of differential expression. Gene expression levels were normalized to fragments per kilobase of transcript per million mapped reads. Significance was determined using a threshold of *P* < 0.05 and a log2 fold change of ≥1.5, as analyzed with Hiplot Enhanced MA v0.1.0, for differential expression. GO and KEGG pathway analyses were performed to explore the biological implications of the DEGs. KEGG pathway analysis was used to connect DEGs with higher-level systemic functions across cells, species, and ecosystems, providing deeper insights into the molecular response networks of protein-coding genes. These analyses were further enriched and interpreted using the Database for Annotation, Visualization, and Integrated Discovery (DAVID) Bioinformatics Resources 6.8.

Deconvolution analysis was used to ascertain metabolic shifts within the four specific cell types—microglial cells, neural stem cells, astrocytes, and neuroblasts–in both mock-, and CVA10-P8-infected brains, leveraging our proteomics data set. Initially, we identified cell-specific markers for the diverse brain cell types from the “CellMarker” database and proteins involved in “Metabolism” pathways from the KEGG database (47). Subsequently, we determined pairwise Spearman correlation coefficients between the expression levels of brain cell markers and metabolic proteins. Finally, protein pairs exhibiting a correlation coefficient＞0.8 were employed to generate a protein–protein interaction network, facilitated by the use of Cytoscape software v3.8.2 (48).

### Flow cytometry analysis

The 14-day-old ICR mice were inoculated via p.o. with 10^6^ TCID_50_ of CVA10-P8. At 7 dpi, the control and CVA10-P8 infected mice (*n* = 7) were euthanized by carbon dioxide. Subsequently, the brain, spleen, and lung tissues are harvested to create single-cell suspensions (25, 49). The antibody used in fluorescence-activated cell sorting analysis was purchased from Biolegend Inc., USA: PerCP/Cyanine5.5 anti-mouse Ly-6C (#128012), APC anti-mouse Ly-6G (#127614), BV711-conjugated CD4 antibody (No. 100549), biotin anti-CD8 antibody (No. 100704), Brilliant Violet 421TM anti-mouse F4/80 (#123132), APC/Cyanine7 anti-mouse CD45 (#147718), FITC anti-mouse/human CD11b (#101206), and Zombie Aqua Fixable Viability Kit (#423102). All cytometry data were collected using CytExpert software 2.4 on a CytoFLEX SRT. Data were analyzed using CytExpert software 2.4 and FlowJo 10.8.1.

### Statistical analysis

All statistical analyses were performed with GraphPad Prism 8.0 (GraphPad Software), and data were presented as the mean ± SD. Each experiment was performed independently. The log-rank (Mantel–Cox) test was used to compare survival among various mouse groups. Differences in tissue viral titers in mice were determined using the one-way analysis of variance test, with *P* values < 0.05 deemed significant. ^**^P < 0.01; ^***^P < 0.001; ^****^P < 0.0001; ns, no significant difference.

## Data Availability

All data related to this research are listed in the main text or supplemental material and are available from the author (Jichen Li) upon reasonable request. All sequencing data in this study are available in the Sequence Read Archive (SRA) database under the accession numbers PRJNA1254215.

## SUPPLEMENTAL MATERIAL

Supplemental figures jvi.00937-25-s0001.docx Figures S1 and S2.

ASM does not own the copyrights to Supplemental Material that may be linked to, or accessed through, an article. The authors have granted ASM a non-exclusive, world-wide license to publish the Supplemental Material files. Please contact the corresponding author directly for reuse.
