# Supplementary material for: Pathological characteristics of a murine oral coxsackievirus A10 infection model
Source: J Virol. 2025 Jul 1;99(7):e00937-25. doi: 10.1128/jvi.00937-25 (PMC12282057; doi:10.1128/jvi.00937-25)

**Fig. S1. Espript Representation of Sequence Alignment.** Amino acid differences between the CVA10 parental strain and the CVA10 adapted strain are marked in red (A-D).

**
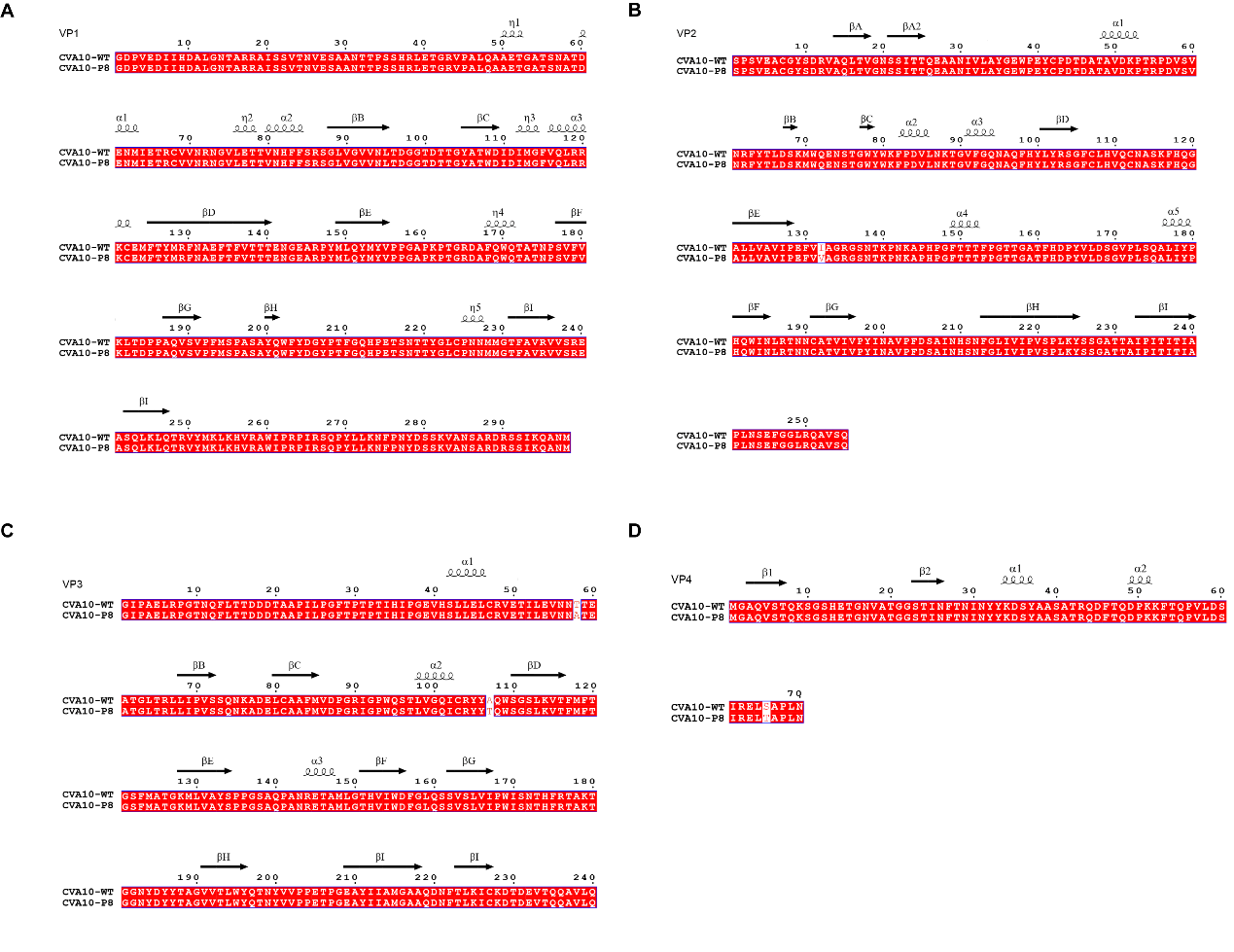
**

**Fig. S2. Utilization of KREMEN-1 by the CVA10 parental strain and the CVA10 adapted strain.** Cytopathic effect caused by CVA10 parental strain and CVA10 adapted strain in non-susceptible cell lines (SK-N-SH) transfected with murine KREMEN-1 (A). The viral titers of the CVA10 adapted strain in SK-N-SH cells at different time points were higher than those of the CVA10 parental strain (B). ^**^*P* < 0.01; ^***^*P* < 0.001; ^****^*P* < 0.0001; ns: Not significant


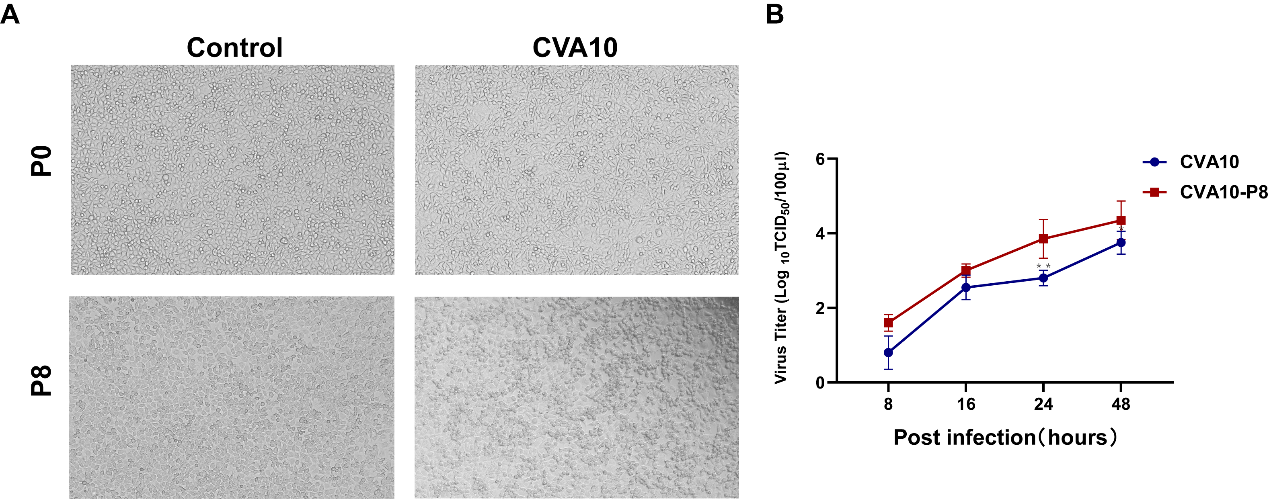

Supplement: Supplemental figures — Figures S1 and S2. [file jvi.00937-25-s0001.docx]
